# Skin Cancer Recognition Using Unified Deep Convolutional Neural Networks

**DOI:** 10.3390/cancers16071246

**Published:** 2024-03-22

**Authors:** Nasser A. AlSadhan, Shatha Ali Alamri, Mohamed Maher Ben Ismail, Ouiem Bchir

**Affiliations:** 1 Computer Science Department, College of Computer and Information Sciences, King Saud University, Riyadh 12372, Saudi Arabia; mbenismail@ksu.edu.sa (M.M.B.I.); obchir@ksu.edu.sa (O.B.); 2 Faculty of Computers and Information Technology, University of Tabuk, Tabuk 47512, Saudi Arabia

**Keywords:** cancer recognition, pattern recognition, CAD systems

## Abstract

**Simple Summary:**

According to the World Fund for Research on Cancer, skin cancer is one of the most common cancers. The early diagnosis of skin cancer lesions plays an essential role in the patient’s recovery. Nevertheless, recognizing skin cancer and differentiating it from benign skin lesions is a challenging task for dermatologists due to the visual similarities of benign nevi, seborrheic keratoses, and malignant melanomas. In this context, image-based skin lesion recognition systems have appeared as a solution to recognize these lesions and therefore reduce the number of biopsy procedures. This research investigated the performance of the latest versions of the You Only Look Once (YOLO) deep learning models. Unlike classification-based solutions, the proposed YOLO-based approach locates the skin lesions and categorizes them into the predefined classes. The experiments were conducted using 2750 images from the publicly accessible International Skin Imaging Collaboration (ISIC) dataset.

**Abstract:**

The incidence of skin cancer is rising globally, posing a significant public health threat. An early and accurate diagnosis is crucial for patient prognoses. However, discriminating between malignant melanoma and benign lesions, such as nevi and keratoses, remains a challenging task due to their visual similarities. Image-based recognition systems offer a promising solution to aid dermatologists and potentially reduce unnecessary biopsies. This research investigated the performance of four unified convolutional neural networks, namely, YOLOv3, YOLOv4, YOLOv5, and YOLOv7, in classifying skin lesions. Each model was trained on a benchmark dataset, and the obtained performances were compared based on lesion localization, classification accuracy, and inference time. In particular, YOLOv7 achieved superior performance with an Intersection over Union (IoU) of 86.3%, a mean Average Precision (mAP) of 75.4%, an F1-measure of 80%, and an inference time of 0.32 s per image. These findings demonstrated the potential of YOLOv7 as a valuable tool for aiding dermatologists in early skin cancer diagnosis and potentially reducing unnecessary biopsies.

## 1. Introduction

Skin cancer represents a global public health crisis with an ever-increasing incidence. It stands as the 19th most common cancer globally, with a disconcerting upward trend [1]. Skin cancer weighs heavily on white-skinned individuals of both sexes, particularly in the United States, where the number of diagnosed cases has skyrocketed in recent decades [2,3]. While preventative measures like UV exposure reduction and screening programs have been implemented, the global prevalence of skin cancer continues its relentless ascent [4]. In fact, an early diagnosis of skin cancer is not merely a matter of statistics. It holds the key to extending life expectancy and improving treatment outcomes. However, the timely and accurate identification of skin cancer remains a challenging, mainly due to the diverse complexity and rapid spread of certain cancer types [5]. In addition, the limitations of traditional diagnostic methods, such as dermoscopy, has further exacerbated this issue. In particular, despite the valuable assistance offered by dermoscopy, its subjectivity, time-consuming procedures, and diagnosis subjectivity have limited its widespread use and promoted the investigation of alternative approaches [6]. On the other hand, coupled spectroscopic and imaging techniques, such as real-time Raman spectroscopy, have shown promise in conjunction with conventional approaches [7]. However, their ability to accurately discriminate between malignant and benign lesions, particularly those with overlapping characteristics, such as malignant melanoma and seborrheic keratosis, remains below expectation [8]. Additionally, the intra-class variations in terms of size, color, and form make the identification problem even more acute [9].

The transformative power of Machine Learning (ML), particularly deep learning, has emerged as a beacon of hope for diverse challenges in the medical field. In particular, ML techniques have revolutionized skin cancer identification through rapid, cost-effective, and accurate diagnoses that couple image classification techniques with deep learning models [10].

One should note that the motivation for this research stems from the profound impact an early diagnosis can have on patients. In fact, early detection offers a critical window of opportunity to (i) improve the prognosis, (ii) significantly enhance the likelihood of successful treatment, and (iii) ultimately save human lives [11]. Recent advancements in YOLO models have represented a potentially transformative alternative for skin cancer detection [12]. In particular, these models are ideally suited for real-time applications due to their computational efficiency, which makes them a promising avenue for rapid and accessible diagnoses.

This research investigated the broader landscape of Machine Learning (ML) and deep learning (DL) in medical applications, particularly in Computer-Aided Diagnosis (CAD) systems. In particular, the proposed YOLO model-based system aligns with the paradigm shift toward deep learning, and here, we demonstrate its potential for skin cancer recognition. Specifically, this study focused on:

▪ Analyzing the state-of-the-art deep learning techniques employed for skin cancer lesion recognition;
▪ Conducting a comprehensive evaluation of the latest YOLO models, namely, YOLOv3, YOLOv4, YOLOv5, and YOLOv7, in terms of their performance and computational efficiency;
▪ Designing and implementing a YOLO-based approach for accurate skin cancer lesion recognition, focusing on the identification of “Malignant Melanoma”, “Benign Nevus”, and “Seborrheic Keratosis” lesions;
▪ Assessing the performance of the proposed system in comparison to the existing methods, using established metrics such as accuracy, sensitivity, specificity, and computational time.

Accordingly, this article addresses the limitations of the current skin cancer recognition methods through the proposed YOLO-based approach. In fact, no existing study has empirically compared YOLOv3, YOLOv4, YOLOv5, and YOLOv7 in classifying skin cancer cases. Moreover, this article represents the first research to investigate YOLOv5 and YOLOv7 for skin lesion recognition. Furthermore, data augmentation was considered for improving the generalization of the designed YOLO model-based approach.

The rest of this manuscript is organized as follows: Section 2 surveys the relevant literature, while Section 3 depicts the proposed skin cancer recognition approach. The experiments and discussion of the obtained results are reported in Section 4. Finally, Section 5 concludes the article.

## 2. Literature Review

In this section, we survey the state-of-the-art deep learning approaches used in the localization, classification, and recognition of skin lesions. First, we will focus on the deep learning methods adopted for skin lesion localization [13]. These methods, often employed as preprocessing steps, aim to identify potentially cancerous regions within an image. In addition, the various architectures and strategies used for accurate localization are outlined in order to highlight their strengths and limitations. Secondly, we will delve into deep learning techniques for classifying skin lesions. In particular, diverse classification models and their ability to distinguish between benign and malignant lesions based on extracted features are explored. Moreover, the performance of different network architectures and training strategies are investigated. Finally, we will address the development of deep learning models for the simultaneous localization and recognition of skin lesions. In fact, these powerful “one-shot” approaches eliminate the need for separate localization and classification steps, potentially saving time and resources. Specifically, we will examine the design principles and performance of such models, offering a perspective on their future in clinical settings.

Deep learning-based approaches rely on object detection models, particularly YOLOv3 [14,15,16,17] for skin lesion localization. Similarly, faster R-CNN was used in [18] to address the skin lesion detection problem. One should mention that most methods target melanoma localization [15,18], while others focus on four common skin cancer types [16,17]. Moreover, the performance measures vary, with some evaluating segmentation post-localization [15,18] and others directly assessing localization accuracy [17]. Notably, these approaches leverage the localization capabilities of deep learning recognition models. Table 1 summarizes the state-of-the-art studies using deep learning-based approaches for localization purposes. 

Other studies primarily tackle the binary classification problem (melanoma vs. benign) using CNNs [20], pre-trained models [21], and ensemble methods [22]. Multi-class classification (e.g., melanoma, common nevus, atypical nevus) was also addressed in [23], [24]. Specifically, AlexNet and DenseNet201 were explored to overcome the skin lesion detection challenges. Recently, YOLO-based approaches have been adapted for combined localization and classification tasks [25,26]. Table 2 summarizes the state-of-the-art studies that exploited deep learning-based approaches for classification purposes.

Accordingly, deep learning has gained traction for skin lesion recognition. Namely, YOLO [14,33,34,35] and Faster R-CNN [19] models were exploited to address these object detection and classification tasks. One should note that YOLOv2 [33] outperformed other YOLO variants in binary classification tasks (e.g., melanoma vs. benign) [11,36]. On the other hand, multi-class recognition (e.g., multiple cancer types) has been explored using YOLOv3 [37]. Moreover, Faster R-CNN has also been applied for melanoma and actinic keratosis recognition [38,39]. Table 3 summarizes the YOLO deep learning-based approaches that have been employed to recognize skin lesions.

## 3. Methodology

An early and accurate diagnosis is crucial for curing skin cancer pathologies. Conventional manual approaches have proven to be costly and time-consuming. This promoted the need for automated intelligent systems. In particular, image processing and deep learning have been introduced as promising alternatives for automated lesion recognition, potentially saving time, costs, and lives.

This research provides a comprehensive comparison between YOLOv3, YOLOv4, YOLOv5, and YOLOv7 models in the context of skin cancer detection. Specifically, the performances of these models in classifying cancers cases, namely melanomas, nevi, and seborrheic keratosis, were investigated.

### 3.1. YOLOv3-Based Model

**Figure 1:**
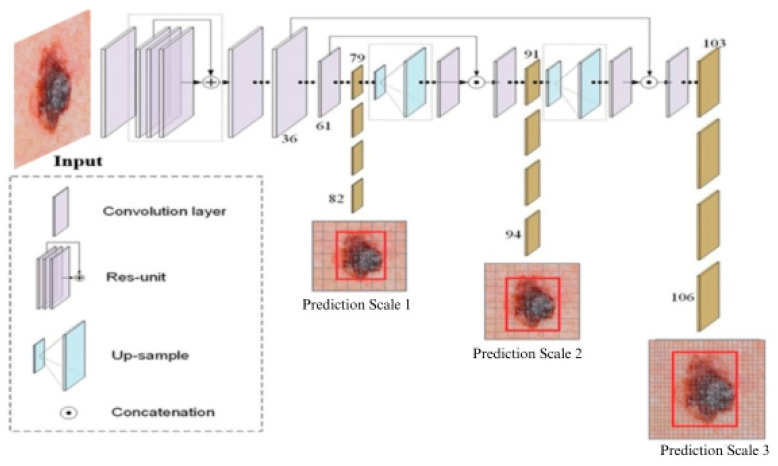
The proposed YOLOv3 architecture.

The proposed YOLOv3 model leverages a pre-trained 53-layer Darknet53 [14] backbone for feature extraction, followed by an additional 53 layers dedicated to recognition, resulting in a total of 106 convolutional layers. This architecture incorporates a Feature-Pyramid Network (FPN) [42] as its neck, which utilizes bottom–up and top–down pathways to extract multi-scale feature maps. The final predictions are made by the YOLO layer, located in the head. A key strength of the proposed YOLOv3 is its ability to perform detections at three different scales, addressing the historical limitation of small object detection in previous YOLO versions. Figure 1 depicts the YOLOv3 architecture that was designed and implemented in this research.

In fact, the considered YOLOv3 loss function combines both binary cross-entropy loss and logistic regression for classification and object confidence prediction, respectively. In addition, class scores are predicted using logistic regression. Note that the classes exceeding a pre-defined threshold are only assigned to a bounding box.

### 3.2. YOLOv4-Based Model

**Figure 2:**
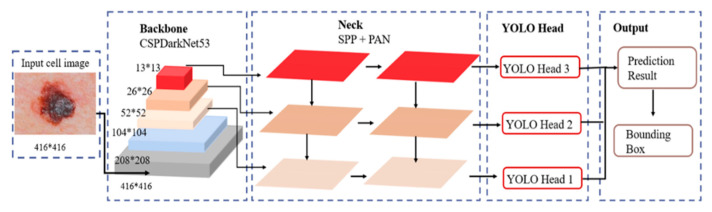
The proposed YOLOv4 architecture.

The proposed YOLOv4 architecture was built upon YOLOv3. Specifically, a Cross Stage Partial Network (CSPNet) [43] was coupled with the Darknet model to form a novel CSPDarknet53 backbone for feature extraction. The resulting DenseNet-inspired [44] convolution architecture addresses the gradient vanishing problem, optimizes the backpropagation, and eliminates processing bottlenecks. This yields improved learning and generalization capabilities. Specifically, the proposed YOLOv4 architecture, shown in Figure 2, involves three sequential blocks, the (i) backbone: CSPDarknet53 extracts features from the input image; (ii) neck: Spatial Pyramid Pooling (SPP) [45] and Path Aggregation Network (PANet) [46] modules expand the receptive field and refine features from the backbone; and (iii) head: basic YOLO layers that generate the final recognition results.

In addition, YOLOv4 employs the Bag of Freebies (BoF) [47] and Bag of Specials (BoS) [35] techniques for optimization. BoF enhances the detector accuracy without increasing inference costs. It employs a Complete IoU (CIoU) loss, a drop block regularization, and diverse augmentation techniques to improve the model generalization. On the other hand, BoS involves plugins and post-processing modules, such as Mish activation, DIoU-NMS [48], and modified PANet, to enhance the object detection accuracy and maintain an acceptable inference cost.

### 3.3. YOLOv5-Based Model

**Figure 3:**
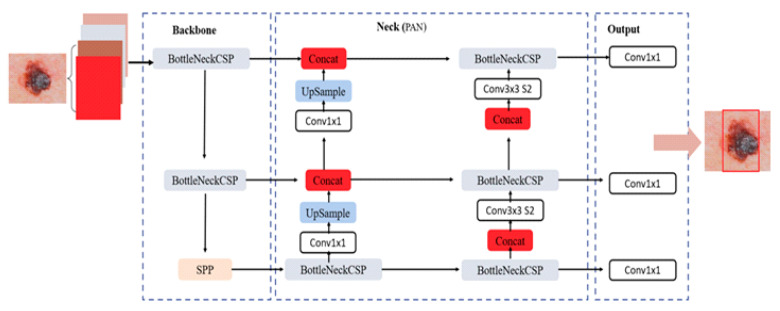
The proposed YOLOv5 architecture.

The proposed YOLOv5 architecture distinguishes itself from YOLOv3 and YOLOv4 through key architectural choices. Specifically, YOLOv5 exhibits a Focus structure, which reduces the model size and addresses gradient redundancy. This yields a faster inference and improved accuracy. Unlike YOLOv3 and YOLOv4 which enclose three separate YOLO layers, YOLOv5 includes one single YOLO layer in the head. This simplification reduces model complexity while maintaining effective multi-scale prediction through three distinct feature maps. One should mention that both YOLOv4 and YOLOv5 utilize a Path Aggregation Network (PANet) [46] in the neck block. Figure 3 illustrates the main blocks of the considered YOLOv5 architecture. It can be seen that the input image is conveyed through the CSPDarknet53 backbone for feature extraction, followed by Spatial Pyramid Pooling (SPP) [45] to generate features at different scales. The extracted features are then fed into PANet for refinement and aggregation. Finally, the single YOLO layer in the head produces the final recognition results.

### 3.4. YOLOv7-Based Model

The proposed YOLOv7 architecture inherits a groundbreaking advancement in real-time object detection [49]. Like its predecessors, YOLOv7 adheres to the traditional backbone–neck–head architecture. To ensure optimal inference speed, YOLOv7 leverages Extended Efficiency Layer Aggregation Networks (E-ELANs) [50] as its final layer aggregation method. An E-ELAN, an enhanced version of the ELAN computational block, was meticulously designed considering the memory requirements, input/output channel ratios, and gradient propagation distances.

In fact, the considered YOLOv7 introduces a novel multi-head structure, incorporating a lead head for final predictions and an auxiliary head that assists in training in intermediate layers. Additionally, it employs a compound scaling strategy that scales the network depth and width while concatenating layers to balance speed and accuracy. In contrast to a scaled YOLOv4 architecture, YOLOv7 backbones are trained exclusively on the MS COCO dataset, opting against ImageNet pre-trained backbones. A noteworthy innovation in YOLOv7 is re-parameterization, a new “bag of freebies” technique that enhances model performance without increasing training costs. When it is applied after training, it further improves the inference results. Notably, YOLOv7 surpasses previous object detectors in both speed and accuracy, solidifying its position at the forefront of the field [49].

### 3.5. Proposed Skin Lesion Recognition Approach

**Figure 4:**
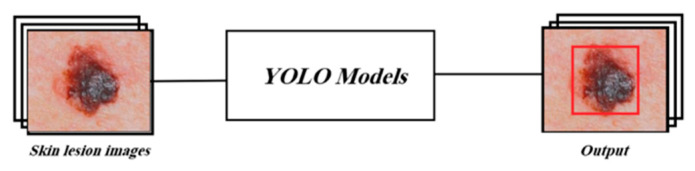
YOLO models’ training framework.

This section outlines the proposed approach to compare the performances of YOLOv3, YOLOv4, YOLOv5, and YOLOv7 in recognizing “Malignant Melanoma” (MM), “Benign Nevus” (BN), and “Seborrheic Keratosis” (SK) skin lesions. Specifically, the four considered models are trained as depicted in Figure 4. Each YOLO model is fed with images representing skin lesions. The labels of these images are also conveyed to the recognition systems.

**Figure 5:**
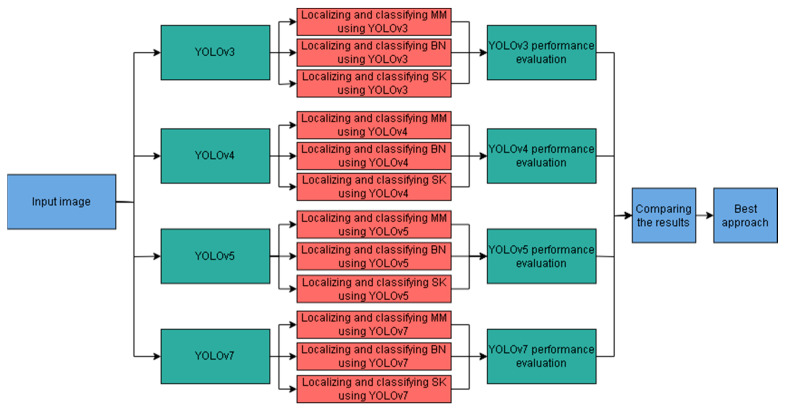
Methodology to design YOLO-based recognition system for skin lesions.

These labels consist of the type of the lesion, Malignant Melanoma (MM), Seborrheic Keratosis (SK), or Benign Nevus (BN), in addition to their bounding boxes’ details. Namely, the upper left corner coordinates, the width, and the height of the box are provided. In addition to training YOLO models, the network hyperparameters are tuned using the pre-defined validation set. The methodology depicted in Figure 5 was adopted to determine the best-performing YOLO version. More specifically, the performances of the considered YOLO models were assessed using the test set in terms of object classification, object localization, and computation time for recognition.

**Figure 6:**
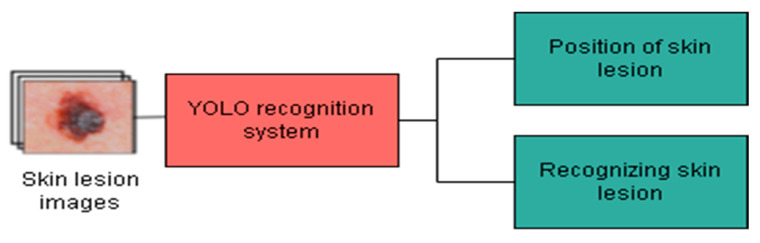
Proposed system architecture.

Then, two data augmentation strategies were adopted. The first one was applied to all training instances without allocating a specific proportion for each class. In other words, the data were increased without taking the data balance into account. In contrast, the second data augmentation uses a certain percentage from each class to accomplish a balance between the classes. The latter was employed to design the proposed system, as illustrated in Figure 6.

The subsequent sections detail the experimental setup, results, and comparisons, providing insights into the proposed model’s performance.

## 4. Experiments

The conducted experiments relied on the International Skin Imaging Collaboration (ISIC) 2017 dataset [13] from the International Skin Imaging Collaboration (ISIC) archive’s “Skin Lesion Analysis Towards Melanoma Detection” challenge. Comprising 2750 dermoscopy images, the dataset exhibits high intra-class variability in texture, color, and size. The images are labeled as “Malignant Melanoma” (MM), “Seborrheic Keratosis” (SK), or “Benign Nevus” (BN) and are distributed across a training set (2000 images), validation set (150 images), and test set (600 images). Their distribution with respect to each class is reported in Table 4. The images were labeled by dermatology experts and their sized range from 540 × 576 pixels to 4499 × 6748 pixels. The details of the considered dataset are depicted in Table 4.

**Figure 7:**
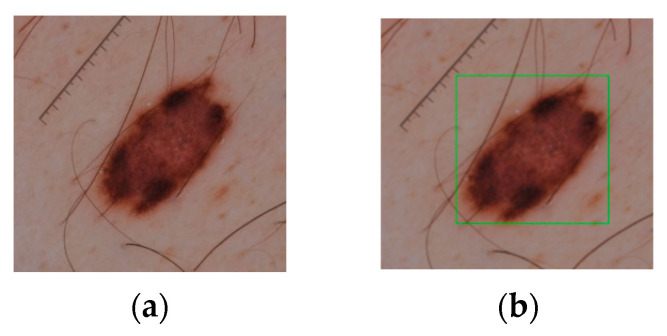
Sample (**a**) dataset image and (**b**) the corresponding bounding box.

The dataset images were annotated by expert dermatologists. Moreover, as shown in Figure 7, the ground truth required for the training of YOLO models was provided as the details of the bounding boxes surrounding the lesion of interest, along with the corresponding class labels.

### 4.1. Performance Measures

Four key metrics were used to evaluate the recognition performance and speed of YOLOv3, YOLOv4, YOLOv5, and YOLOv7 in detecting skin lesions:

1. **Intersection over Union (IoU):** Quantifies the spatial overlap between predicted and ground truth bounding boxes (0–1 scale; 1 = perfect overlap), which is shown in Equation (1).
2. **Average Precision (AP):** Integrates IoU, precision, and recall across various confidence thresholds, summarizing the localization and classification accuracy for each class, which is shown in Equation (2).
3. **Mean Average Precision (mAP):** Averages AP across all classes, providing a single overall performance indicator, which is shown in Equation (3).
4. **F1-measure:** Harmonic mean of precision and recall, offering a balanced view of model performance for each class, which is shown in Equation (4).

These standard metrics strike a balance between accuracy, detail, and conciseness, allowing for an effective comparison of model performances in skin lesion recognition. We considered both spatial accuracy (IoU) and classification accuracy (AP, mAP, F1-measure), while accounting for the impact of confidence thresholds (AP). Additionally, we measured the running time to evaluate the feasibility of real-world application.

(1) $$\mathrm{IoU}=\frac{\mathrm{Area}\;\mathrm{of}\;\mathrm{Intersection}\;}{\mathrm{Area}\;\mathrm{of}\;\mathrm{Union}}$$

(2) $$\mathrm{AP}=\int_{0}^{1}p\left(r\right)dr\;$$

(3) $$\mathrm{mAP}\;=\frac{1}{N}\overset{N}{\underset{i=1}{\sum }}{\mathrm{AP}}_{c}$$

(4) $$F1-\mathrm{Measure}=\frac{2*\mathrm{Recall}*\mathrm{Precision}}{\mathrm{Recall}+\mathrm{Precision}}$$

### 4.2. Results

To comprehensively evaluate the performance of YOLOv3, YOLOv4, YOLOv5, and YOLOv7 in skin lesion recognition, we conducted four distinct experiments. Each experiment employed a specific model with a defined configuration, ensuring a fair and systematic comparison. Table 5 shows the detailed configuration adopted for each considered model. Specifically, the settings of the Learning rate (Lr), the Momentum (M), and the number of batches (B) are reported.

**Figure 8:**
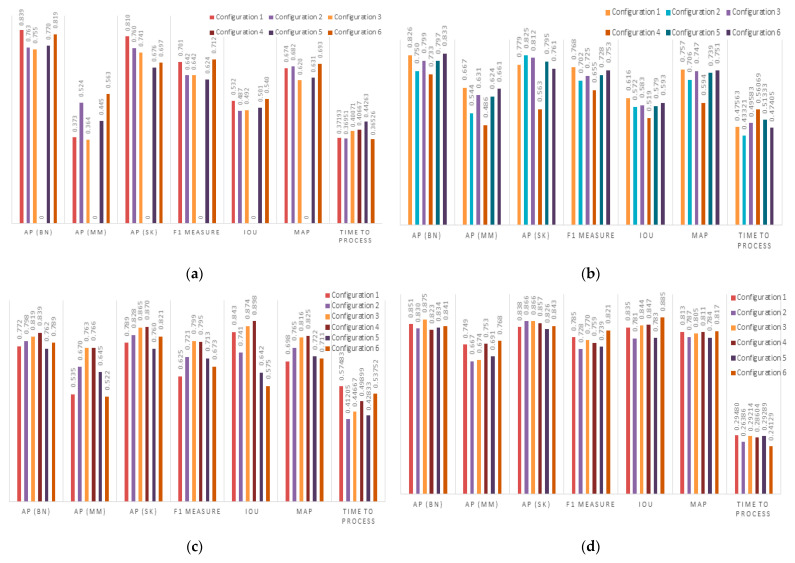
Performance results obtained using (**a**) YOLOv3, (**b**) YOLOv4, (**c**) YOLOv5, and (**d**) YOLOv7.

Figure 8 summarizes the performance of each configuration and for each model on the dataset across the chosen metrics. Additionally, the running time per image is reported to assess the feasibility of real-world application.

To further validate the findings from the initial experiment, we evaluated the performance of each model with its best configuration on the held-out test and validation sets. This assessment provides a more rigorous evaluation of generalizability and robustness using unseen data. The achieved results are detailed in Table 6. The best result for each metric is shown in bold.

**Figure 9:**
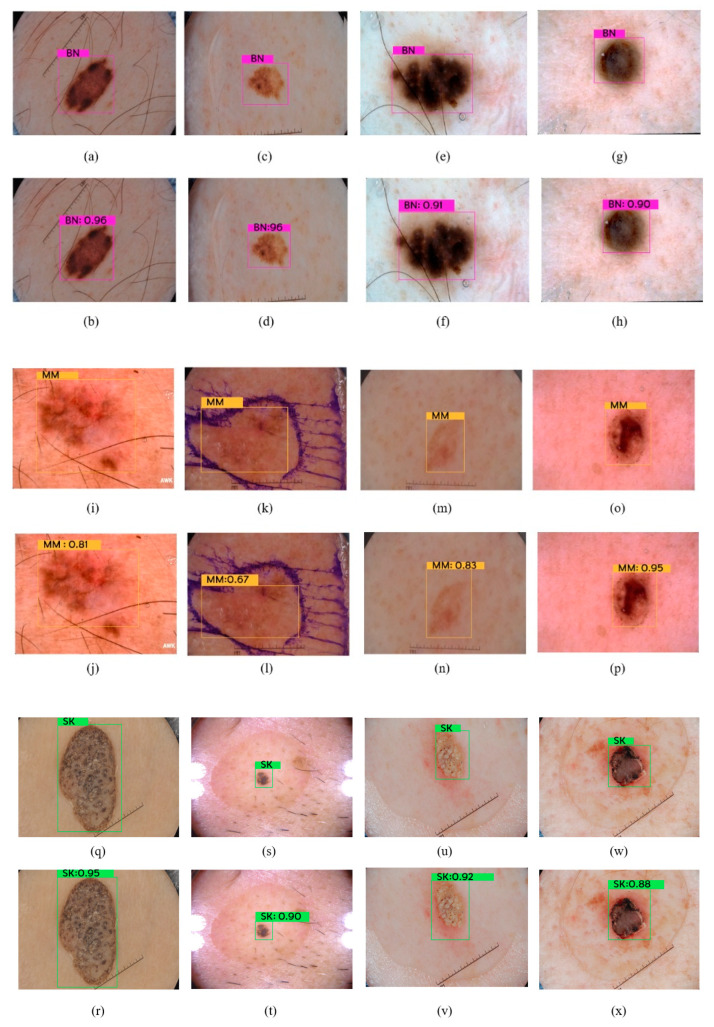
Sample detections of (**a**–**h**): benign nevi (BN), (**i**–**p**): malignant melanoma (MM), and (**q**–**x**): seborrheic keratosis (SK) lesions using YOLOv7.

To further illustrate the capabilities of the models for skin lesion recognition, we showcase the detection results of YOLOv7 on different lesion types. In particular, Figure 9 shows sample detections of benign nevi (BN), malignant melanomas (MM), and seborrheic keratosis (SK). It can be seen that YOLOv7 accurately located and identified the lesions of interest. Moreover, the generated bounding boxes precisely surround the lesions. This showcases YOLOv7’s strong spatial localization capabilities.

### 4.3. Discussion

Table 6 demonstrates YOLOv7’s significant edge over YOLOv3 and YOLOv4 in recognizing the three considered lesion types, namely BN, MM, and SK. This finding is consolidated by superior mAP, IoU, and F1-measure values compared to those of both YOLOv3 and YOLOv4. While YOLOv5 exhibited slightly better performance for benign nevus detection and a marginally higher IoU, YOLOv7 surpassed it in terms of inference time, F1-measures, and mAP. Notably, YOLOv7 boasted a significantly faster processing time per image (0.31 s vs. 0.51 s for YOLOv5), making it more suitable for real-time applications. Additionally, YOLOv7 demonstrated consistently higher F1-measures and mAP across all lesion categories, indicating superior overall accuracy and balanced performance.

These advancements can be attributed to YOLOv7’s architectural improvements: a novel model scaling component that optimizes factors like layer count, channel count, feature pyramid stages, and input image resolution for optimal performance, and a multi-head structure that further guides the training process, contributing to the model’s superior accuracy and efficiency.

The experimental results reveal that while YOLOv5 and YOLOv7 showcased comparable detection performances, YOLOv7 reigns supreme in terms of inference time, F1-measures, and mAP. Consequently, YOLOv7 served as the chosen model for the proposed approach due to its combined advantages in accuracy, speed, and robustness.

However, it is important to acknowledge that YOLOv7 exhibited a lower AP for malignant melanoma compared to benign nevi and seborrheic keratosis. This can be attributed to two factors: the visual similarities that malignant melanoma shares with the other categories, potentially leading to misclassification, and the data imbalance within the dataset, where the number of malignant melanoma instances was smaller than the number of instances of the other two categories.

To address this challenge, data augmentation was employed on the training set. This technique artificially expands the malignant melanoma data, providing the model with diverse training examples and potentially mitigating classification errors for this category.

To further enhance YOLOv7’s performance, we employed data augmentation to artificially expand the training set through a variety of transformations including rotation, flipping, cropping, scaling, translation, shearing, and color adjustments. Specifically, we implemented two strategies: first, applying augmentation to the entire training set, generating a pool of 20,000 images and second, recognizing the class imbalance, we balanced the data by keeping all augmented malignant melanoma (MM) images while adding only 20% and 70% more augmented benign nevus (BN) and seborrheic keratosis (SK) images, respectively, resulting in a roughly equal class representation, which is shown in Table 7.

Table 8 shows that data augmentation consistently boosted YOLOv7’s performance, with the exception of processing time, with balanced augmentation yielding the most pronounced impact. Notably, MM’s Average Precision (AP) soared from 64.9% to 68.4% (compared to 66.8% with unbalanced augmentation), showcasing the effectiveness of targeted data augmentation in addressing class imbalances.

Table 9 reports the performance achieved using the YOLOv7 model with those obtained using relevant state-of-the-art approaches. Namely, we compared YOLOv7 with a Convolutional Neural Network (CNN) with 69 layers introduced in [51]. Furthermore, the results obtained using the MobileNet-V2, Xception, and InceptionResNet-V2 models proposed in [52] were also considered in this comparison study.

As can be seen in Table 9, YOLOv7 consistently outperformed the state-of-the-art models according to the evaluation metrics. Notably, YOLOv7 yielded the highest AP, F1-score, IoU, and mAP values. One can also note that InceptionResNet-V2 model outperformed Xception model. Moreover, the CNN-based approach in [51] was overtaken by all considered models.

## 5. Conclusions and Future Work

The escalating prevalence of skin cancer demands urgent attention, as it poses a global public health threat. Early detection plays a crucial role in successful treatment, highlighting the need for accurate and efficient diagnostic methods. While traditional physician assessments remain the standard, their inherent limitations like subjectivity and time constraints necessitate alternative approaches. This project tackled this challenge by proposing an image-based system powered by deep learning to automatically recognize and differentiate cancerous skin lesions from benign ones. We focused on three prevalent lesions—malignant melanoma, benign nevi, and seborrheic keratosis.

To lay a solid foundation, we explored the background of these lesions, outlining their distinctive visual characteristics. We then delved into supervised learning and deep learning paradigms, specifically examining Convolutional Neural Networks (CNNs), ResNets, Darknets, and YOLO models. Furthermore, we investigated recent deep learning-based approaches for skin lesion recognition, focusing on those involving localization, classification, and recognition. The existing works using YOLOv3 and YOLOv4 demonstrated promising results, but a gap existed in comparing all four YOLO models—YOLOv3, YOLOv4, YOLOv5, and YOLOv7—for this specific task. Moreover, YOLOv5 and YOLOv7 remained unexplored in the domain of skin lesion recognition.

This paper bridged this gap by comprehensively evaluating and comparing the performance of all four YOLO models in automatically recognizing the three targeted lesions from the ISIC 2017 dataset. The results revealed YOLOv7 as the most effective model, further improving its performance by utilizing a balanced augmented training set. This highlights the value of targeted data augmentation in addressing class imbalances.

Looking ahead, exciting future research opportunities lie in exploring semantic segmentation deep learning approaches like DeepLabv3 for more nuanced lesion recognition along with the newer ISIC 2019 [53] and ISIC 2020 [54] datasets. Additionally, investigating the potential of Generative Adversarial Networks (GANs) for data generation presents a promising avenue for expanding and enriching training datasets, potentially unlocking further advancements in skin cancer diagnosis through the power of deep learning.

## Figures and Tables

**Table 1:** Summary of deep learning-based approaches adopted for skin lesion localization.

Performance					Skin Cancer	Model	Ref.
**Specificity**			**Sensitivity**		Melanoma	YOLOv3 [14]	[15]
97.05			97.33				
97.5			97.5				
97.02			97.97				
**Intersection Over Union (IOU)**			**Accuracy**		Benign, Melanoma, Seborrheic Keratosis, Atypical Nevi,	YOLOv3 [14]	[16]
90			94.4				
86			96				
**Mean Box IOU**			**Mean Average Precision (mAP)**		Benign, Melanoma, Seborrheic Keratosis, Atypical Nevi,	YOLOv3 [14]	[17]
79.03			91.85				
**Jaccard**	**Dice**	**Specificity**	**Sensitivity**	**Accuracy**	Melanoma	Faster R-CNN [19]	[18]
80.9	91.5	97.3	96.8	95.9			
89.1	95.2	98.8	97.5	97.9			
80.9	94.7	98.1	97.2	97.1			

**Table 2:** Summary of deep learning-based approaches for classifying skin lesions.

Performance					Skin Cancer	Model	Ref.
**NPV**	**PPV**	**Specificity**	**Sensitivity**	**Accuracy**	Normal Melanoma	Deep Believe Network [27]	[28]
94.12	86.76	89.7	91.18	92.65			
**F1-Score**	**Precision**		**Recall**	**Accuracy**	Benign Malignant	CNN [29]	[20]
83.25	83.25		84	89.5			
**Accuracy**					Benign Malignant	VGG-16 [30]	[21]
78							
**Precision**	**Specificity**		**Sensitivity**	**Accuracy**	Melanoma, Nevi, Atypical nevi	AlexNET [31]	[23]
97.73	98.93		98.33	98.61			
**Avg. F-measure**	**Avg. Precision**	**Avg. Recall**	**Accuracy**	**Architecture**	Actinic Keratosis, Basal Cell Carcinoma, Benign Keratosis, Dermatofibroma, Nevi, Melanoma, Vascular	DenseNet2 [32]	[24]
91.26	92.03	90.5	94.52	**Plain DenseNet2**			
85.05	85.3	84.8	91.73	**Two-level DenseNet2**			
**AUC**		**Precision**	**Recall**	**Accuracy**	Benign Malignant	YOLOv2 [33]	[25]
0.95		85	88	94			
**AUC**	**Precision**	**Specificity**	**Sensitivity**	**Accuracy**	Melanoma Non-Melanoma	YOLOv3 [14]	[26]
0.99	97.5	99.37	97.5	99			
0.99	97.44	99.38	97.44	99			
0.99	94.64	98.13	94.22	97.11			

**Table 3:** Summary of YOLO deep learning-based approaches for recognizing skin lesions.

Performance					Skin Cancer	Model	Ref.
**mAP**			**Model**		Benign Malignant	YOLOv1 [34] YOLOv2 [33] YOLOv3 [14]	[36]
37			YOLOv1				
83			YOLOv2				
77			YOLOv3				
**AUC**		**Specificity**	**Sensitivity**	**Accuracy**	Melanoma Non-Melanoma	YOLOv2 [33]	[11]
91		85.9	86.35	86			
**mAP**	**F1-Score**	**Precision**	**Recall**	**Accuracy**	Benign Malignant	YOLOv4 [35]	[40]
89.34	85	81	89	94.04			
**Precision**		**Recall**	**Accuracy**	**Skin Cancer**	Basal Cell Carcinomas, Bowen’s Disease	YOLOv3 [14]	[41]
91.3		32.8	91.3	**BCC**			
90.9		30.3	90.9	**Bowen’s Disease**			

**Table 4:** Details of ISIC 2017 dataset.

Class Type	Training Set	Validation Set	Testing Set
Malignant Melanoma (MM)	374	30	117
Seborrheic Keratosis (SK)	254	42	90
Benign Nevus (BN)	1372	78	393

**Table 5:** Models’ hyper-parameter settings.

	YOLOv3			YOLOv4			YOLOv5			YOLOv7		
Configuration\Hyperparameter	Lr	*M*	*B*	Lr	*M*	*B*	Lr	*M*	*B*	Lr	*M*	*B*
Configuration 1	0.001	0.937	64	0.001	0.900	64	0.001	0.949	32	0.001	0.937	50
Configuration 2	0.0001	0.949	64	0.0001	0.960	64	0.005	0.937	16	0.0001	0.949	50
Configuration 3	0.001	0.950	16	0.001	0.949	32	0.01	0.937	32	0.001	0.950	32
Configuration 4	0.1	0.990	32	0.01	0.990	32	0.01	0.900	16	0.01	0.949	16
Configuration 5	0.01	0.949	32	0.01	0.937	64	0.001	0.949	64	0.01	0.990	32
Configuration 6	0.005	0.900	64	0.005	0.900	64	0.01	0.950	64	0.005	0.900	60

**Table 6:** Performance achieved using test and validation sets.

	Validation Set							Test Set						
	AP(BN)	AP(MM)	AP(SK)	mAP	IoU	F1	Time to Process (s)	AP(BN)	AP(MM)	AP(SK)	mAP	IoU	F1	Time to Process (s)
YOLOv3	81.9	56.3	69.7	69.3	53.9	69.2	0.36	79.5	42.7	60.5	60.9	50.8	65.0	0.45
YOLOv4	82.6	66.7	77.9	75.7	61.6	76.8	0.47	81.5	52.6	65.4	66.5	60.8	72.0	0.50
YOLOv5	83.9	76.3	**86.7**	**82.5**	**89.8**	79.9	0.49	**81.6**	61.9	74.9	72.8	**87.4**	74.2	0.51
YOLOv7	**84.1**	**76.8**	84.3	81.7	88.5	**82.1**	**0.24**	80.1	**64.9**	**81.3**	**75.4**	86.3	**77.9**	**0.31**

**Table 7:** Class distribution with/without data augmentation.

Class	No Data Augmentation	Data Augmentation
BN	1372	2740
MM	254	2540
SK	374	2610

**Table 8:** Effect of data augmentation on YOLOv7 performance results.

Test Results	Class	AP	F1-Score	IoU	mAP	Processing Time (s)
No data augmentation	BN	80.1	77.9	86.3	75.4	**0.44**
	MM	64.9				
	SK	81.3				
Data augmentation—imbalanced	BN	81.4	78.3	87.5	76.5	0.59
	MM	66.8				
	SK	81.4				
Data augmentation—balanced	BN	**81.9**	**79.6**	**88.7**	**78.0**	0.54
	MM	**68.4**				
	SK	**83.8**				

**Table 9:** Comparison of YOLOv7 performance with relevant state-of-the-art approaches.

Approach	Class	AP	F1-Score	IoU	mAP
CNN from [51]	BN	74.3	73.5	92.7	72.2
	MM	61.7			
	SK	80.5			
MobileNet-V2 from [52]	BN	78.5	74.9	83.1	74.7
	MM	67.4			
	SK	78.1			
Xception from [52]	BN	77.0	76.6	85.9	75.1
	MM	67.3			
	SK	81.0			
InceptionResNet-V2 from [52]	BN	76.8	77.9	86.5	75.4
	MM	67.4			
	SK	82.0			
Proposed YOLOv7 with balanced data augmentation	BN	81.9	79.6	88.7	78.0
	MM	68.4			
	SK	83.8			

## Data Availability

A publicly available dataset was analyzed in this research. This data can be found at https://challenge.isic-archive.com/data/#2017 (5 February 2023).
